# Supplementary material for: Noncommunicable disease risk behaviors and protective factors among children in Samoa: Retrospective trend analysis of global school-based health surveys in 2011 and 2017
Source: PLOS Glob Public Health. 2024 Jun 11;4(6):e0003315. doi: 10.1371/journal.pgph.0003315 (PMC11166286; doi:10.1371/journal.pgph.0003315)
Supplement: S1 Table — (DOCX) [file pgph.0003315.s001.docx]

**S1 Table.** Summary of variables and questions from the Global school-based health survey (GSHS) in Samoa*

| Variable | GSHS Question | Response Options |
| --- | --- | --- |
| **Overweight/Obesity** |  |  |
| Weight | How much do you weigh without your shoes on? | Kilograms |
| Height | How tall are you without your shoes on? | Meters |
| **Dietary behavior** |  |  |
| Ate Fruits | “During the past 30 days, how many times per day did you usually eat fruit, such as pawpaw, ripe banana, coconut, apple, or orange? | 1 = I did not eat fruit during the past 30 days to 7 = 5 or more times per day |
| Ate Vegetables | During the past 30 days, how many times per day did you usually eat vegetables, such as laupele or cabbage? | 1= I did not eat vegetables during the past 30 days to 7 = 5 or more times per day |
| Drank carbonated soft drinks | During the past 30 days, how many times per day did you usually drink carbonated soft drinks, such as Coca-Cola? | 1= I did not drink carbonated soft drinks during the past 30 days to 7= 5 or more times per day |
| Went hungry | During the past 30 days, how often did you go hungry because there was not enough food in your home? | 1 = never to 5 = always |
| **Physical Activity and Sedentary Behaviors** |  |  |
| Physical Activity | During the past 7 days, on how many days were you physically active for a total of at least 60 min per day?” | 1 = 0 days  to 8 = 7 days |
| Sitting | How much time do you spend during a typical or usual day sitting and watching television, playing computer games, talking with friends, or doing other sitting activities such as using your cell phone? | 1 = Less than 1 h per day . . . 3 = 3 to 4 hours per day . . . 6 = 8 or more hours a day |
| Attended physical education classes | During this school year, on how many days did you go to physical education (PE) class each week? | 1= 0 days to 6= 5 or more days |
| **Substance Use** |  |  |
| Smoked cigarettes in the past month | During the past 30 days, on how many days did you smoke cigarettes?” | 1 = 0 days to 7 = all 30 days |
| Smoked other tobacco products in the past month | During the past 30 days, on how many days did you use any other form of tobacco, such as e-cigarettes or chewed smokes?” | 1 = 0 days to 7 = all 30 days |
| Drank any alcohol in the past month | During the past 30 days, on how many days did you have at least one drink containing alcohol? | 1 = 0 days to 7 = All 30 days |
| Drunk in life time | During your life, how many times did you drink so much alcohol that you were really drunk? | 1 = 0 times to 4 = 10 or more times |
| **Oral and Hand Hygiene** |  |  |
| Brushed teeth | During the past 30 days, how many times per day did you usually clean or brush your teeth? | 1= I did not clean or brush my teeth during the past 30 days to 6= 4 or more times per day |
| Wash hands before eating | During the past 30 days, how often did you wash your hands before eating? | 1 = never to 5 = always |
| Wash hands after toilet use | During the past 30 days, how often did you wash your hands after using the toilet or latrine? | 1 = never to 5 = always |
| Wash hands with soap | During the past 30 days, how often did you use soap when washing your hands? | 1 = never to 5 = always |
| **Emotional and Mental Health** |  |  |
| Missed class or school without permission (absenteeism) | During the past 30 days, on how many days did you miss classes or school without permission? | 1= 0 days to 5=10 or more days |
| Bullied | During the past 30 days, on how many days were you bullied? | 1 = 0 days to 7 = All 30 days |
| In physical fight | During the past 12 months, how many times were you in a physical fight? | 1 = 0 times to 8 = 12 or more times |
| Felt lonely | During the past 12 months, how often have you felt lonely? | 1 = never to 5 = always |
| Worried that could not sleep at night | During the past 12 months, how often have you been so worried about something that you could not sleep at night? | 1 = never to 5 = always |
| Had no close friends | How many close friends do you have? | 1=0 to 4=3 or more |
| Considered attempting suicide | “During the past 12 months, did you ever seriously consider attempting suicide?” | 1 = yes, 2 = no |
| Made a plan to attempt suicide | “During the past 12 months, did you make a plan about how you would attempt suicide?” | 1 = yes, 2 = no |
| Attempted suicide in past 12 months | During the past 12 months, how many times did you actually attempt suicide? | 1=0 times to 5=6 or more times |
| **Community protective factors** |  |  |
| Peer support: Other students were kind and helpful | During the past 30 days, how often were most of the students in your school kind and helpful?” | 1 = never to 5 = always |
| Parental/guardian supervision: check homework | “During the past 30 days, how often did your parents or guardians check to see if your homework was done?” | 1 = never to 5 = always |
| Parental connectedness: Parents/guardians understand troubles | “During the past 30 days, how often did your parents or guardians understand your problems and worries?” | 1 = never to 5 = always |
| Parents/guardians know what you do | “During the past 30 days, how often did your parents or guardians really know what you were doing with your free time?” | 1 = never to 5 = always |

* All de-identified datasets, questionnaires, codebooks, and other study documents were downloaded from the WHO NCD microdata repository and CDC website for this analysis (9, 10, 25-27)
