# Supplementary material for: Noncommunicable disease risk behaviors and protective factors among children in Samoa: Retrospective trend analysis of global school-based health surveys in 2011 and 2017
Source: PLOS Glob Public Health. 2024 Jun 11;4(6):e0003315. doi: 10.1371/journal.pgph.0003315 (PMC11166286; doi:10.1371/journal.pgph.0003315)
Supplement: S2 Table — (DOCX) [file pgph.0003315.s002.docx]

**S2 Table.** Health risk behaviors among all students in 2011 and 2017*

|  | 2011 | |  | 2017 | |
| --- | --- | --- | --- | --- | --- |
|  | % | 95% CI |  | % | 95% CI |
| **Overweight/obesity**  (>+1SD from the median for body mass index by age and sex) | 51.98 | 49.14-54.82 |  | 59.20 | 56.75-61.65 |
| **Dietary behavior** |  |  |  |  |  |
| Ate Fruits (2+ times/ day) | 49.94 | 47.82-52.05 |  | 50.92 | 46.97-54.88 |
| Ate Vegetables (3+ times per day) | 38.10 | 35.22-40.99 |  | 34.28 | 31.39-37.17 |
| Drank carbonated soft drinks (1+ times per day) | 54.15 | 51.28-57.02 |  | 63.42 | 60.55-66.29 |
| Went hungry (mostly/always) | 36.15 | 33.42-38.89 |  | 12.54 | 9.07-16.02 |
| **Physical Activity and Sedentary Behaviors** |  |  |  |  |  |
| Active 60+ mins/day for 5+ of past 7 days | 20.90 | 18.73-23.07 |  | 31.20 | 27.90-34.50 |
| Time sitting 3+ hours/day | 39.48 | 35.68-43.28 |  | 27.85 | 22.90-32.79 |
| Attended physical education classes 5+days/week during  school year | 13.80 | 10.54-17.06 |  | 15.77 | 12.10-19.43 |
| Missed class or school without permission (absenteeism) | 55.95 | 50.47-61.43 |  | 37.00 | 33.79-40.22 |
| **Substance Use** |  |  |  |  |  |
| Currently smoked cigarettes in the past month | 36.08 | 29.84-42.31 |  | 10.22 | 8.61-11.82 |
| Currently smoked other tobacco products in the past  month | 40.64 | 34.91-46.37 |  | 8.63 | 6.89-10.38 |
| Drank any alcohol in the past month | 36.49 | 30.30-42.68 |  | 12.14 | 9.57-14.71 |
| Drunk in life time | 37.35 | 31.23-43.47 |  | 8.32 | 5.96-10.68 |
| **Oral and Hand Hygiene** |  |  |  |  |  |
| Brushed teeth (1- times/day) | 81.13 | 77.71-84.56 |  | 95.28 | 93.43-97.12 |
| Wash hands before eating (not always) | 14.98 | 13.18-16.78 |  | 18.79 | 16.52-21.07 |
| Wash hands after toilet use (not always) | 17.91 | 15.26-20.56 |  | 7.28 | 5.50-9.06 |
| Wash hands with soap (not always) | 19.38 | 17.12-21.65 |  | 14.79 | 11.97-17.61 |
| **Emotional and Mental Health** |  |  |  |  |  |
| Bullied | 74.75 | 69.26-80.23 |  | 37.69 | 33.02-42.35 |
| In physical fight | 68.74 | 65.30-72.19 |  | 47.87 | 43.23-52.51 |
| Felt lonely (mostly/always) | 23.61 | 22.00-25.21 |  | 8.99 | 7.65-10.32 |
| Worried that could not sleep at night (mostly/always) | 28.16 | 24.59-31.73 |  | 9.52 | 7.69-11.35 |
| Had no close friends | 16.31 | 14.15-18.47 |  | 10.00 | 8.04-11.96 |
| Seriously considered attempting suicide in the past 12  months | 33.75 | 30.79-36.71 |  | 22.94 | 20.53-25.35 |
| Made plan to attempt suicide in the past 12 months | 40.29 | 35.64-44.95 |  | 22.96 | 20.76-25.17 |
| Attempted suicide in the past 12 months | 62.00 | 54.34-69.66 |  | 23.00 | 19.83-26.17 |
| **Community protective factors (reported as most/always)** |  |  |  |  |  |
| Other students were kind and helpful | 33.79 | 30.21-37.37 |  | 35.80 | 32.41-39.19 |
| Parents/guardians check homework | 41.67 | 39.08-44.25 |  | 47.34 | 42.88-51.81 |
| Parents/guardians understand troubles | 32.64 | 30.09-35.19 |  | 25.11 | 22.01-28.20 |
| Parents/guardians know what you do | 30.35 | 27.83-32.87 |  | 28.51 | 25.45-31.58 |

*Percentages and 95% confidence interval (CI) are weighted and adjusted for the multistage stratified cluster sample design of the survey
