# Supplementary material for: Noncommunicable disease risk behaviors and protective factors among children in Samoa: Retrospective trend analysis of global school-based health surveys in 2011 and 2017
Source: PLOS Glob Public Health. 2024 Jun 11;4(6):e0003315. doi: 10.1371/journal.pgph.0003315 (PMC11166286; doi:10.1371/journal.pgph.0003315)
Supplement: Attachment — Submitted filename: Response to Reviewers PGPH-D-24-00472.docx [file pgph.0003315.s005.docx]

Dear Editor and Reviewers,

We are grateful for your positive feedback and the opportunity to improve the rigor of our work. For the manuscript (Tracking #PGPH-D-24-00472**)**, responses to your individual comments are included below in blue. New changes to the original version are included using track changes in MS word in a marked-up copy, as a separate file labeled 'Revised Manuscript with Track Changes'. An unmarked version of our revised paper without tracked changes is uploaded as a separate file labeled 'Manuscript', as instructed.

Journal Requirements:

1. Please review your reference list to ensure that it is complete and correct. If you have cited papers that have been retracted, please include the rationale for doing so in the manuscript text, or remove these references and replace them with relevant current references. Any changes to the reference list should be mentioned in the rebuttal letter that accompanies your revised manuscript. If you need to cite a retracted article, indicate the article’s retracted status in the References list and also include a citation and full reference for the retraction notice.

Thank you, the reference list has been updated. It is complete and correct.

2. We ask that a manuscript source file is provided at Revision. Please upload your manuscript file as a .doc, .docx, .rtf or .tex.

We provided a .docx file for every manuscript version at revision.

Additional Editor Comments (if provided):

[Note: HTML markup is below. Please do not edit.]

Noted with thanks.

Reviewers' comments:

Reviewer's Responses to Questions

**Comments to the Author**

1. Does this manuscript meet PLOS Global Public Health’s [publication criteria](https://journals.plos.org/globalpublichealth/s/criteria-for-publication)? Is the manuscript technically sound, and do the data support the conclusions? The manuscript must describe methodologically and ethically rigorous research with conclusions that are appropriately drawn based on the data presented.

Reviewer #1: Yes

Reviewer #2: Yes

2. Has the statistical analysis been performed appropriately and rigorously?

Reviewer #1: Yes

Reviewer #2: Yes

3. Have the authors made all data underlying the findings in their manuscript fully available (please refer to the Data Availability Statement at the start of the manuscript PDF file)?

The [PLOS Data policy](https://journals.plos.org/globalpublichealth/s/data-availability) requires authors to make all data underlying the findings described in their manuscript fully available without restriction, with rare exception. The data should be provided as part of the manuscript or its supporting information, or deposited to a public repository. For example, in addition to summary statistics, the data points behind means, medians and variance measures should be available. If there are restrictions on publicly sharing data—e.g. participant privacy or use of data from a third party—those must be specified.

Reviewer #1: Yes

Reviewer #2: Yes

4. Is the manuscript presented in an intelligible fashion and written in standard English?

PLOS Global Public Health does not copyedit accepted manuscripts, so the language in submitted articles must be clear, correct, and unambiguous. Any typographical or grammatical errors should be corrected at revision, so please note any specific errors here.

Reviewer #1: Yes

Reviewer #2: Yes

5. Review Comments to the Author

Please use the space provided to explain your answers to the questions above. You may also include additional comments for the author, including concerns about dual publication, research ethics, or publication ethics. (Please upload your review as an attachment if it exceeds 20,000 characters)

Reviewer #1: This is an interesting paper. The authors have successfully established the need for research, and have focused on a pertinent public health problem. Data analysis is robust and findings have been well interpreted.

We appreciate your supportive comments.

Reviewer #2: attached

We appreciate your constructive feedback and have made changes based on the comments in the attached PDF document.

Title Page Line 2: Were the years 2011 to 2017 or only 2001 and 2017. Please check this ambiguity

Thank you for raising this question. The data were from 2011 and 2017, and we have clarified this information in the manuscript tile, which is now changed to “Noncommunicable disease risk behaviors and protective factors among children in Samoa: Retrospective trend analysis of global school-based health surveys in 2011 and 2017”. Throughout the manuscript, we were careful and updated the wording to reference the data as “in 2011” and “in 2017”, and not ‘between’.

Table 1: 11> and 16<

We appreciate this suggestion and have changed the labels for the age groups to “≤11” and “≥16”, respectively.

6. PLOS authors have the option to publish the peer review history of their article ([what does this mean?](https://journals.plos.org/globalpublichealth/s/editorial-and-peer-review-process#loc-peer-review-history)). If published, this will include your full peer review and any attached files.


**Do you want your identity to be public for this peer review?** If you choose “no”, your identity will remain anonymous but your review may still be made public.


For information about this choice, including consent withdrawal, please see our [Privacy Policy](https://www.plos.org/privacy-policy).

Reviewer #1: No

Reviewer #2: No

[NOTE: If reviewer comments were submitted as an attachment file, they will be attached to this email and accessible via the submission site. Please log into your account, locate the manuscript record, and check for the action link "View Attachments". If this link does not appear, there are no attachment files.]

Thank you for this note, we have responded above to the reviewer’s comments from the attached PDF document in this letter.

While revising your submission, please upload your figure files to the Preflight Analysis and Conversion Engine (PACE) digital diagnostic tool, <https://pacev2.apexcovantage.com/>. PACE helps ensure that figures meet PLOS requirements. To use PACE, you must first register as a user. Registration is free. Then, login and navigate to the UPLOAD tab, where you will find detailed instructions on how to use the tool. If you encounter any issues or have any questions when using PACE, please email PLOS at figures@plos.org. Please note that Supporting Information files do not need this step.

We appreciate this reminder, and the manuscript does not include any figure files. Tables 1-3 are in the manuscript file. S1 Table and S2 Table are included as supporting information.
